# Intraosseous Type 2 Median Nerve Entrapment After Posterior Elbow Dislocation Diagnosed on Ultrasound With MRI and Surgical Correlation

**DOI:** 10.7759/cureus.18606

**Published:** 2021-10-08

**Authors:** Amy C O'Brien, Zoe Teh, Marta Rinaldi, Elsa Lee, Richard Hughes, Ioannis Aktselis, David McKean

**Affiliations:** 1 Radiology Department, Buckinghamshire Healthcare NHS Trust, Aylesbury, GBR; 2 School of Medicine, Cardiff University, Cardiff, GBR; 3 National Spinal Injuries Centre, Buckinghamshire Healthcare NHS Trust, Aylesbury, GBR; 4 Radiology, King's College London, London, GBR; 5 Orthopaedic Surgery Department, Buckinghamshire Healthcare NHS Trust, Aylesbury, GBR

**Keywords:** ultrasound, median neuropathy, magnetic resonance imaging, joint dislocations, elbow

## Abstract

Median nerve entrapment is a rare complication of posterior elbow dislocation and medial epicondyle fracture. In the event of delayed diagnosis, this injury pattern may result in significant and sometimes irreversible nerve damage. As such, a high degree of clinical suspicion and early imaging is indicated in patients with persistent nerve deficits following reduction of elbow dislocation.

Here, a case of intraosseous type 2 median nerve entrapment that was diagnosed on ultrasound in an eight-year-old patient following ulnohumeral dislocation is discussed. This article reviews the key imaging findings of median nerve entrapment and discusses the subsequent MRI and surgical findings of this rare condition.

## Introduction

Posterior elbow dislocation can result in median nerve entrapment as a rare complication. In the event of delayed diagnosis, this injury pattern may result in significant and sometimes irreversible nerve damage. In the clinic context of persistent nerve deficits after elbow dislocation, this pathology must be suspected and early imaging must be obtained.

In this case report, a case of intraosseous type 2 median nerve entrapment in an eight-year-old patient, diagnosed on ultrasound, following ulnohumeral dislocation is discussed. This article reviews the key imaging findings of median nerve entrapment and discusses the subsequent MRI and surgical findings of this rare condition. Medial epicondyle fractures are common, accounting for approximately 11-20% of paediatric elbow fractures [1,2]. While several mechanisms have been implicated, posterior elbow dislocation is the most common, accounting for two-thirds of cases [3]. Other mechanisms include falling on an outstretched hand while the elbow is in full extension, which can result in traction on the flexor-pronator muscle group and can cause an avulsion injury. Rarely, a direct trauma can cause a medial epicondylar fracture. There are numerous complications relating to ulnohumeral dislocation, including elbow stiffness, damage to local neurovascular structures of the elbow, and reduced mobility or range of motion.

In one series, 6.3% of elbow dislocations resulted in injury to the ulnar nerve [4]. Less commonly, median nerve injuries can occur. Entrapment of both the median nerve and the anterior interosseous nerve (its motor branch) has been reported due to an osseous bridge formation occurring as a result of a healing medial epicondylar fracture [5-8]. While entrapped osseous fragments post elbow dislocation are often radiologically and clinically apparent, an entrapped nerve can easily be overlooked in the immediate postoperative period. If the patient has persistent neurological symptoms following an elbow dislocation, nerve entrapment should be strongly considered [6,7,9]. When it comes to diagnosing nerve entrapment, ultrasound may be considered a first-line imaging modality as it is widely available and readily accessible. In addition, it is also less likely to require sedation in the paediatric population. However, there have been several reports of median nerve entrapment which have relied primarily on MRI for final diagnosis [10,11]. It is important to note that many of the cases in the literature describe a delay in the diagnosis of median nerve entrapment; in some cases, imaging was only performed years after the injury when symptoms did not improve [9].

Median nerve entrapment is an indication for surgical intervention, and early diagnosis is critical to prevent irreversible neurological injury [10]. Type 1 entrapment occurs due to an avulsed medial epicondyle when the median nerve is trapped between the olecranon and trochlea. Type 2 entrapment occurs not when the nerve is within the joint but when it is entrapped within the healed medial epicondyle fracture. Type 3 entrapment occurs when the nerve becomes entrapped and kinked between the olecranon and distal humerus within the joint. Type 4 entrapment occurs when the nerve is entrapped both within the joint and within the healed fracture. Type 4 may have a worse prognosis than type 3 due to double entrapment sites [11].

## Case presentation

**Figure 1:**
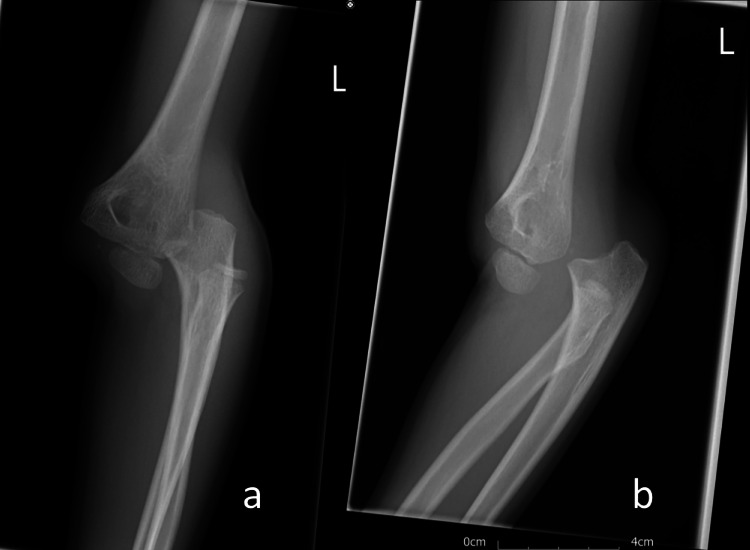
(a) Anteroposterior and (b) lateral radiographs at the time of the initial injury demonstrating a posterior elbow dislocation.

**Figure 2:**
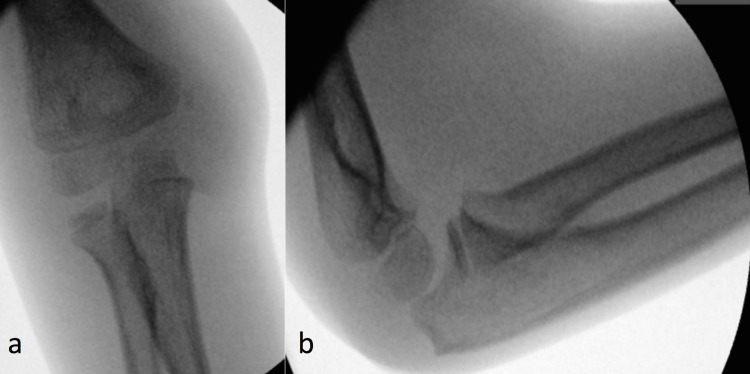
Intraoperative (a) anteroposterior and (b) lateral radiographs demonstrating satisfactory reduction of the posterior elbow dislocation, with no other osseous injury evident at this time. Undisplaced medial ossification centre noted.

An eight-year-old male presented to the emergency department in July 2020. He had fallen from a bicycle and landed on his left (non-dominant) arm with a visible deformity of the elbow. It was initially reported that his arm was cold and pulseless. However, by the time he attended the Accident and Emergency Department, his capillary return was reasonable. Plain film examination confirmed the diagnosis of posterohumeral dislocation (Figure 1), and the patient was manipulated under general anaesthesia in the theatre (Figure 2).

**Figure 3:**
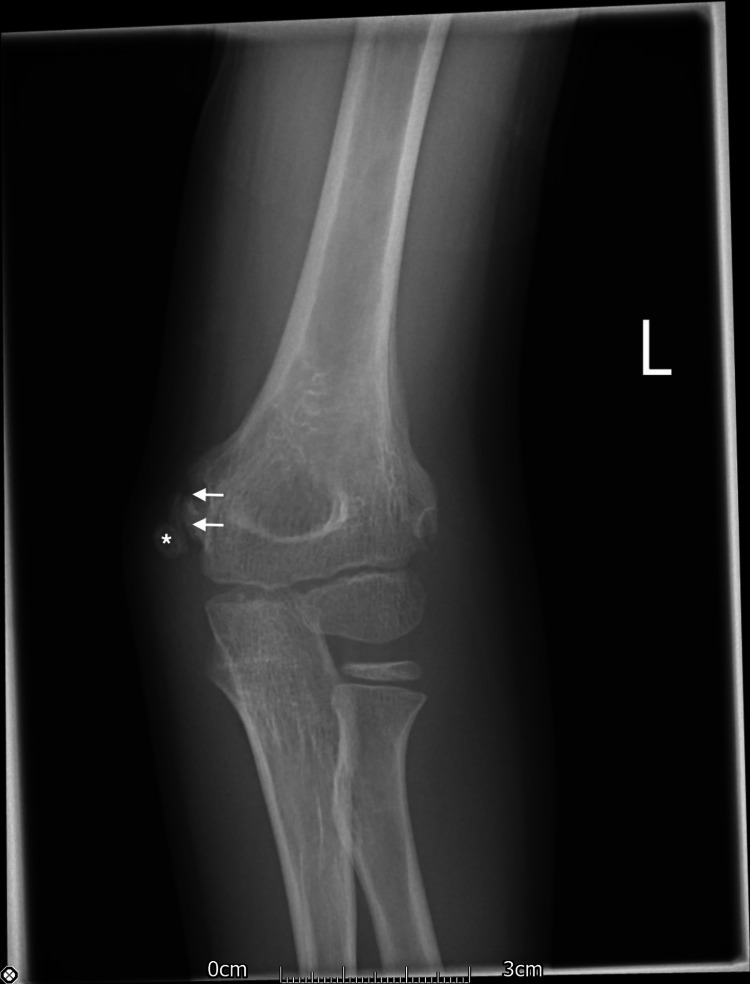
Six-month postoperative anteroposterior radiograph demonstrating osseous bridging of the medial epicondyle (asterisk). There is impression of an osseous tunnel formed along the medial epicondyle (arrows). The patient had persistent neurological symptoms and paraesthesia in the median nerve distribution.

Preoperatively, it was noted that he had numbness in the distribution of the median nerve. Postoperatively, his pulses were restored and the arm was well perfused and warm. However, his sensory and motor median nerve symptoms persisted. The patient was put in an above elbow back slab and discharged the following day. At the one-week follow-up, it was noted that his median nerve symptoms persisted, with weakness of the flexor digitorum profundus (FDP) and the flexor pollicis longus (FPL) of the index finger. However, there was evidence of some recovery with active flexion of the FDP to the index finger, but the FPL was not recorded as showing evidence of reinnervation at that time. These findings were found to persist on clinic assessment one month after the injury. The patient continued with weekly physiotherapy until December 2020 when thenar wasting and persistent difficulty moving the fingers in his left hand, although without sensory symptoms, became apparent. On examination, he was found to have wasting of the thenar musculature and weakness of the abductor pollicis brevis (APB). He also had weakness of the FPL and the FDP. Median nerve sensation was intact but altered, and he had normal ulnar and radial nerve function. Plain film examination demonstrated abnormal ossification of the medial ossification centre (Figure 3).

**Figure 4:**
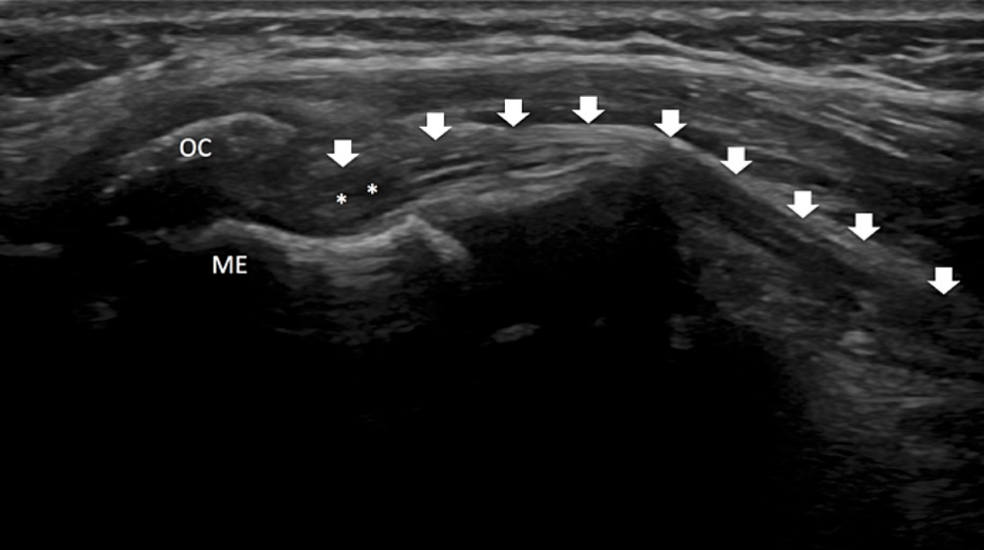
Six-month postoperative ultrasound examination demonstrating the median nerve (arrows) which is seen to deviate deep to the medial ossification centre at the level of the medial epicondyle where it is obscured by acoustic shadowing. There is focal hypoechogenicity of the median nerve just distal to this osseous tunnel consistent with intraneural oedema. The appearances are consistent with intraosseous type 2 median nerve entrapment.

The patient had persistent neurological symptoms and paraesthesia in the median nerve distribution, and an ultrasound was requested to assess the median nerve. To confirm the median nerve entrapment, ultrasound was performed and the ulnar nerve was assessed. The median nerve appeared normal in echogenicity in the short and long axes (Figure 4) at the level of the distal arm. However, as it passed deep to the bony landmark of the medial epicondyle ossification centre, it was seen to appear kinked and deviated posteriorly. Although the nerve at this point was obscured by acoustic shadowing, it was visualized proximally as it exits the osseous tunnel. It demonstrated hypoechoic echotexture at this point, raising the possibility of intraneural oedema. This was best assessed on long-axis images in the proximal forearm (Figure 4).

The ulnar nerve was also assessed on ultrasound. It appeared normal in echogenicity, size, and morphology along its course. Urgent MRI was performed to fully characterise the course of the median nerve. MRI of the elbow confirmed the abnormal course of the medial nerve, with an abrupt posterior course at the level of the medial epicondyle, as well as an impression of local osseous encasement before coursing anteriorly into the forearm (Video 1). The nerve demonstrated increased signal on short inversion time inversion recovery-weighted images, but appeared contiguous with significant thickening of the nerve just proximal to the osseous tunnel and distally within the forearm. The ulnar nerve appeared normal in signal and position on MRI. There was minor oedema of the FDP muscle body, suggestive of denervation changes (Video 1).

**Video 1:** Six-month postoperative MRI examination demonstrating the abnormal tract of the median nerve which courses within an osseous tunnel and makes an acute turn before it exits the tunnel distally.

Surgical exploration and operative mobilization of the medial epicondyle was performed with release of the median nerve from the osseous tunnel. The median nerve was seen distally and proximally during the operation. It was traced to an osseous tunnel between the medial epicondyle and an apophyseal fracture fragment. The immature bony callus was removed and the medial epicondyle was successfully mobilized. The median nerve was released from the osseous tunnel. A significant proportion of the median nerve was found to have been damaged and, after freshening of the edges, end-to-end anastomosis without tension was performed.

At his most recent postoperative follow-up visit six months postoperatively, he had recovered median nerve sensory and motor function. He now had 4+/5 motor strength of the FPL and FDP to the index finger. He had no further paraesthesia and had a normal sensory examination.

## Discussion

In the setting of elbow dislocations, medial epicondyle fractures are not uncommon. However, elbow dislocation can lead to median nerve entrapment in rare circumstances in the paediatric population [4]. In this case, persistent neurological symptoms, despite apparent satisfactory surgical reduction of the dislocation, led to further investigation, with type 2 entrapment of the median nerve demonstrated on ultrasound, further characterised on MRI, and confirmed during surgical exploration.

Type 1 entrapment occurs due to an avulsed medial epicondyle when the median nerve is trapped between the olecranon and trochlea. Type 2 entrapment, as in this case, occurs not when the nerve is within the joint, but rather when entrapped within the healed medial epicondyle fracture. Type 3 entrapment occurs when the nerve becomes entrapped and kinked between the olecranon and distal humerus within the joint. Type 4 entrapment occurs when the nerve is entrapped both within the joint and within the healed fracture. Type 4 may have a worse prognosis than type 3 due to double entrapment sites [12].

Our case highlights the importance of a detailed neurological examination following elbow dislocation. Valuable information can be gained from early imaging, which may provide insight into the presence, type, and extent of nerve entrapment or injury. While postoperative radiographs can evaluate the osseous structures, they do not provide any information regarding nerve entrapment. Electrophysiological testing is useful for nerve function; however, it is invasive and may be difficult to perform in children. Ultrasound neurography has numerous advantages. In addition to availability, accessibility, and low cost, ultrasound can be performed in the paediatric population without sedation. Ultrasound can assess the echogenicity of nerves and identify soft tissue or osseous causes of nerve compression. It allows the ability to compare with the contralateral side, as well as dynamic imaging capabilities, as demonstrated in this case [8,13]. MRI can evaluate nerve damage based on signal characteristics and can demonstrate secondary signs of nerve injury, including intramuscular oedema or atrophy which is useful in a specific nerve distribution [14]. It is important to remember that in the immediate postoperative period, posttraumatic soft tissue signal abnormalities may obscure the evaluation of small peripheral nerves, and secondary signs will not be present at this early stage.

## Conclusions

Median nerve entrapment within a healing medial epicondyle fracture is a rare but potentially serious complication of elbow dislocation in paediatric patients. Early diagnosis is vital for mobilisation of the nerve and a positive outcome. Delayed diagnosis can, unfortunately, lead to poor outcomes and permanent neurological deficits. In the setting of persistent neuropathic symptoms following elbow dislocation, nerve entrapment should be considered and prompt imaging investigations should be obtained using ultrasound or MRI.
